# How does performance-based financing affect the availability of essential medicines in Cameroon? A qualitative study

**DOI:** 10.1093/heapol/czz084

**Published:** 2019-12-09

**Authors:** Isidore Sieleunou, Anne-Marie Turcotte-Tremblay, Manuela De Allegri, Jean-Claude Taptué Fotso, Habakkuk Azinyui Yumo, Denise Magne Tamga, Valéry Ridde

**Affiliations:** 1 Research for Development International, Opposite Fokou Mendong, Yaoundé 30 883, Cameroon; 2 University of Montreal Public Health Research Institute, 7101 Avenue du Parc, Room 3060, Montreal, QC H3N 1X9, Canada; 3 Social and Preventive Medicine, School of Public Health, University of Montreal, 7101 Avenue du Parc, Montreal, QC H3N 1X9, Canada; 4 Medical Faculty and University Hospital, Heidelberg Institute of Global Health, Heidelberg University, INF 130.3, Heidelberg 69120, Germany; 5 World Bank, Office of Yaoundé, Nouvelle Route Bastos, Yaoundé 1128, Cameroon; 6 Cellule Technique Nationale FBP, Tsinga, Yaoundé 237, Cameroon; 7 IRD (French Institute for Research on Sustainable Development), CEPED (IRD-Université Paris Descartes), Universités Paris Sorbonne Cités, ERL INSERM SAGESUD, 45 rue des Saints Pères, Paris 75006, France

**Keywords:** Performance-based financing, essential medicines, essential drugs, Cameroon

## Abstract

Performance-based financing (PBF) is being implemented across low- and middle-income countries to improve the availability and quality of health services, including medicines. Although a few studies have examined the effects of PBF on the availability of essential medicines (EMs) in low- and middle-income countries, there is limited knowledge of the mechanisms underlying these effects. Our research aimed to explore how PBF in Cameroon influenced the availability of EMs, and to understand the pathways leading to the experiential dimension related with the observed changes. The design was an exploratory qualitative study. Data were collected through in-depth interviews, using semi-structured questionnaires. Key informants were selected using purposive sampling. The respondents (*n* = 55) included health services managers, healthcare providers, health authorities, regional drugs store managers and community members. All interviews were recorded, transcribed and analysed using qualitative data analysis software. Thematic analysis was performed. Our findings suggest that the PBF programme improved the perceived availability of EMs in three regions in Cameroon. The change in availability of EMs experienced by stakeholders resulted from several pathways, including the greater autonomy of facilities, the enforced regulation from the district medical team, the greater accountability of the pharmacy attendant and supply system liberalization. However, a sequence of challenges, including delays in PBF payments, limited autonomy, lack of leadership and contextual factors such as remoteness or difficulty in access, was perceived to hinder the capacity to yield optimal changes, resulting in heterogeneity in performance between health facilities. The participants raised concerns regarding the quality control of drugs, the inequalities between facilities and the fragmentation of the drug management system. The study highlights that some specific dimensions of PBF, such as pharmacy autonomy and the liberalization of drugs supply systems, need to be supported by equity interventions, reinforced regulation and measures to ensure the quality of drugs at all levels.

**Key Messages**

- Performance-based financing (PBF) led to an improvement of the perceived availability of essential medicines in Cameroon through several pathways: more autonomy of health facilities (HFs), enforced regulation from the district medical team, greater accountability of the pharmacy attendant and supply system liberalization;
- Many challenges, including delays in PBF payments, limited autonomy, leadership and contextual factors, hindered the capacity to yield optimal changes;
- Attention should be paid to reinforce regulation and ensure the quality of drugs once the supply system is open to private wholesalers;
- PBF interventions should promote equity interventions when granting pharmacy autonomy to HFs to avoid worsening pre-existing inequalities.

## Introduction

Access to safe, effective, quality and affordable medicines is one of the specific objectives included in the Sustainable Development Goals (World Health Assembly, 2016). This access is a key step towards achieving Universal Health Coverage. However, the availability of essential medicines (EMs) is still suboptimal in low- and middle-income countries (Mahmić-Kaknjo *et al.*, 2018). Studies in low- and middle-income countries indicate that the average availability of generic medicines included in national essential medicine lists in the public sector ranges from 27% to 44% (Cameron *et al.*, 2009; Robertson *et al.*, 2009). More recent research in 23 low- and middle-income countries did not find considerable progress, indicating the median availability of EMs at only 40% (Bazargani *et al.*, 2014). In Cameroon, research conducted by Van Der Geest already in the earlier 1980s showed that the availability of medicines at primary health centres was around 50% (Van der Geest, 1982), and a further study over a decade later did not report any progress (Chana Chapchet, 2009).

The availability of EMs in low- and middle-income countries is influenced by a variety of complex, underlying factors such as inadequate financing, weak facility management, regulatory issues, lengthy procurement processes and poor logistics (Quick, 2003; Quick *et al.*, 2005; Zaidi *et al.*, 2013; Van den Ham *et al.*, 2011; Bigdeli *et al.*, 2014; Ewen *et al.*, 2017).

Innovative financing strategies have the potential to increase the availability of medicines (Pronyk *et al.*, 2016). Amongst these strategies, performance-based financing (PBF) is regarded as a promising health system reform that improves service provision, including the quantity and quality of medicines available (Meessen *et al.*, 2007; Basinga *et al.*, 2011; Fritsche *et al.*, 2014). PBF is a financing instrument that involves financial incentives being rewarded to health service entities, such as health districts and health facilities (HFs), for achieving a predefined set of objectives (Oxman and Fretheim, 2009). Recent studies have described the approach as a reform package that goes beyond mere performance-based payments to include a separation of functions (purchaser, provider and regulator), the strategic management and planning of health services, and the participation of local stakeholders in the decision-making process at the health-facility level (Soeters, 2015; Renmans *et al.*, 2016).

In 2008, with World Bank funding, the government of Cameroon launched PBF in 26 health districts in 4 of its 10 regions (Littoral, Northwest, Southwest and East), covering a total population of 3 million inhabitants. Alongside the implementation of the project, the World Bank conducted an impact evaluation (IE) in three out of the four regions (East, Northwest and Southwest) (Sieleunou *et al.*, 2017).

Findings from the quantitative IE showed, on the one hand, a significant impact of PBF on the availability of family planning methods, and on the other hand, no evidence of an impact on other EM groups, including malaria drugs, general medicines and vaccines (De Walque *et al.*, 2017). Mostly quantitative, these studies did not look at what mechanisms PBF puts in place to facilitate the availability of EMs and how these mechanisms interact with the overall context of the health system to promote or to hinder the availability of EMs. In addition, the PBF experience in Cameroon provides a unique opportunity to further our understanding of the effect of PBF on the availability of EMs in the context of drug supply system liberalization, in which the State has lost its monopoly and the system is opened to private actors.

Drawing on in-depth interviews with 55 key informants in four regions of Cameroon, this article aims to explore if and how the PBF scheme in Cameroon influenced the availability of EMs. The study is complementary to the quantitative IE of the PBF programme in Cameroon but was not part of it, hence both were totally independent.

## Contextual background

### PBF and drug procurement in Cameroon

Before the introduction of the PBF programme in Cameroon, the drug supply chain and management system were quasi-monopolistic. The public national EMs supply system in Cameroon was co-ordinated around a single player at the national level: the National Essential Drugs and Disposables Procurement Centre. At the regional level, there were two types of entities in charge of drugs supply and distribution: the Regional Funds for Health Promotion (RFHP) and the Regional Supply Pharmaceuticals Centres.

In 2008, the government launched its PBF scheme. The beneficiaries were public, private for-profit and faith-based facilities. The PBF intervention entailed a shift from an input-based to an output-based financing of healthcare services by introducing performance contracts between the performance purchasing agency (PPA) and single facilities. These contracts focused on the complementary package of activities for hospitals (i.e. a package of healthcare services as per country’s health policy), and the minimum package of activities for health centres (i.e. a package of promotional, preventive and curative health services as per country’s health policy). Performance contracts regulated both performance payments from the PPA to the facilities and performance bonuses that facilities could distribute to their staff, which could not be >60% of the total PBF income. The PPA signed quarterly contracts with health districts both to evaluate the technical quality (i.e. the physical and organizational elements of healthcare) of health centres and also, with the Regional Delegation of Public Health, to (1) organize peer evaluation of hospitals, and (2) carry out enhanced supervision of the health districts’ activities. Moreover, the PPA signed quarterly contracts with community-based organizations to conduct evaluations at the community level. Contracts targeted priority services (respectively, 23 and 25 indicators for the minimum and the complementary packages), mostly related to maternal and child health, and operated according to a fee-for-service basis with additional adjustment made for quality of service delivery (AEDES, 2012). Facilities received payment after verification by the PPA staff of the quantity and quality of services delivered. At least in principle, facilities retained autonomy over the use of PBF payments, based on priorities identified in their business plans, including the provision of performance or retention bonuses to health workers and the purchase of equipment and drugs. In principle, facilities could procure EMs from government-approved distributors and retail outlets and were not obliged to purchase them from any single source. There was no quantitative indicator specifically targeting the quality or the availability of EMs. However, modules in the qualitative evaluation of facilities focused on (1) the availability of vaccines, tracer drugs, oral and injectable contraceptives, and (2) drugs and supplies management. Of the 168 points on the quality checklist, the evaluation of EMs accounted for 35 points, representing about 21% of the total technical quality evaluation (Supplementary Appendix S1).

The opening of the drug procurement system to private actors was a new practice in Cameroon. Although the Regional Supply Pharmaceuticals Centres and the RFHP were the sole organization mandated to supply and distribute drugs to public facilities at the regional level, the PBF system brought other players into this drug supply system. In addition, for the sake of the sustainability, ownership and integration of the PBF intervention into the health system, the RFHP in each region became the PPA (Sieleunou *et al.*, 2017). In the context of Cameroon, the provision of healthcare was already liberalized in the mid-1980s through a mix of public and private providers. The liberalization of the drug supply system for the public health sector was introduced with the PBF project and was limited to the project area. Hence, other public HFs not included in the PBF project continued to rely on the single national public EMs procurement actor. The facilities’ ability to procure inputs locally rather than from central supply management is one of the key elements of PBF schemes recommended by PBF promoters. According to the PBF toolkit’s principle, ‘drugs and medical consumables should be procured from certified distributors, which can include, but are not restricted to, the central medical stores’ (Fritsche *et al.*, 2014, p. 142). Liberalization was intended to stimulate improvements in drug supply by introducing market forces, and hence competition, in a setting traditionally dominated by exclusive government provision.

### Intervention theory

**Figure 1:**
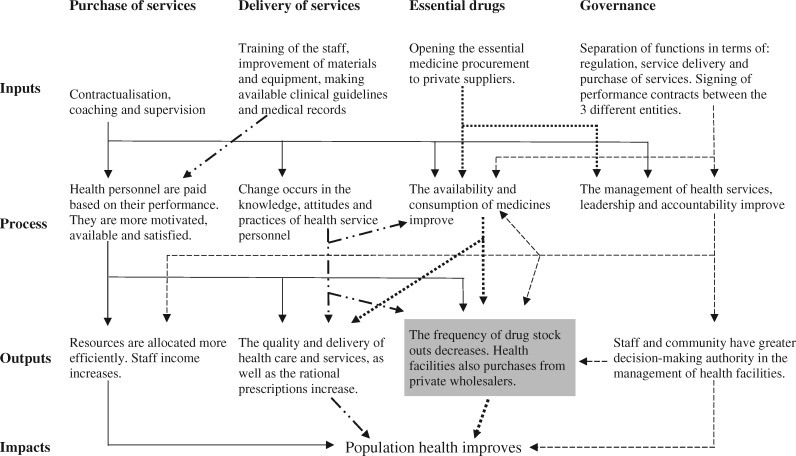
Intervention theory.

The intervention theory on EM is presented in Figure 1, where the results upon which our research focused are presented in grey. The theory was developed during an evaluability assessment phase (Leviton *et al.*, 2010; Dunet *et al.*, 2013) based on: (1) a previous research project on PBF in Burkina Faso conducted by members of our team (Ridde *et al.*, 2014); (2) a documentary review in Cameroon; and (3) five interviews conducted during our exploratory mission.

PBF in Cameroon placed an emphasis on four areas: (1) the purchase of services; (2) services delivery; (3) drugs and medical consumables; and (4) governance. The ‘purchase of services’ refers to the financial incentives that the purchasing agency gave to facilities in exchange for the services provided and the ‘service delivery’ refers to the actual healthcare services that the providers gave to patients. These areas of intervention were supported by two functions: the strengthening of the supervision of HFs and their management autonomy. For the EMs-related management autonomy, PBF provided facility managers total control over pharmacy management (financial, human and equipment). PBF supported the four above-mentioned areas with the coaching and supervision of health personnel, payments for health services and personnel tied to qualitative and quantitative performances, the opening of drug procurement systems to private actors, and the separation of functions (regulator, payer and provider).

PBF was expected to improve EM availability in HFs due to changes applied at three levels: supply system, regulation and/or facility management (World Bank, 2011; AEDES, 2012). Within this specific setting and in relation to our study, availability is further defined as the relationship between the type and quantity of EMs needed as defined by national guidelines and the type and quantity of these EMs available at the facility level (Management Sciences for Health, 2012).

The PPA was expected to sign performance contracts with the Regional Public Health Delegation for, among other things, the maintenance of the quality of the EMs and the drawing up of the list of accredited wholesalers. HFs could then use the list to select a supplier of choice. With the liberalization, competition between different actors was expected to result in a more balanced sale price from suppliers and an increased the availability and quantity of EMs. HFs were expected to use the additional funding from the PBF programme to strengthen the infrastructures and logistics (e.g. buying of motorbikes, refurbishing/construction of new buildings …) that could support the purchase, collection, transportation and storage of EMs. The intervention explicitly mentioned the need for regulators to focus more on their core functions, and performance indicators were defined in relation to more formative supervisions and coaching for staff, and more quality assurance of EMs (Supplementary Appendices S2 and S3).

## Methods

### Study design

We employed an exploratory qualitative study (Creswell, 2014). Exploratory research is designed to understand a phenomenon or a process on which little or no previous research has been conducted (Caelli *et al.*, 2003; Merriam, 2007). The strength of this research design lies in its facilitation in providing insights into the social aspects of participants being investigated, and provides rich data depicting their experiences (Denzin and Lincoln, 2017).

### Study settings

Cameroon is divided into a three-tier administrative system including 10 regions, 58 divisions and 360 sub-divisions. The population of Cameroon was estimated to be 22 218 000 inhabitants in 2015. The health system in Cameroon is decentralized and organized into central, regional and district levels.

At the time of the study, the PBF programme was operational in 26 health districts located in four regions (14 health districts in the East, 4 in the Littoral, 4 in the Northwest and 4 in the Southwest) covering a total of 661 HFs (185 HFs in the East, 308 in the Littoral, 86 in the Northwest and 82 in the Southwest; World Bank, 2013). The study was conducted in all the four regions implementing the PBF programme. Below is a brief description of these regions:

1. With 109 002 km^2^, the East is the largest region in Cameroon and the most sparsely populated with a total estimated population of 1 110 000 inhabitants in 2015. The region is divided into 14 health districts, all implementing PBF. The region was governed through the French direct rule administration until the early 1960s. Although the East region is blessed with natural resources, its population remains one of the poorest in the country, and the region’s rurality is also one of the most important in Cameroon.
2. The Littoral region, located at the seacoast, has a surface area of 20 248 km^2^ and a population of 3 355 000 as of 2015. The region has 19 health districts, of which four were implementing PBF. The region was governed through the French direct rule administration until the early 1960s. The Littoral region is the wealthiest region in the country, with the largest sea port and the main industrial area of Central Africa. It also has large plantations of banana and cocoa.
3. Located in the western highlands of Cameroon, the Northwest region has a surface area of 17 812 km^2^ and an estimated total population of 2 716 000 inhabitants for 2015. The population is unevenly distributed among 19 health districts, of which four were implementing PBF. The Northwest constituted one of the two English-speaking regions of Cameroon. Up to the early 1960s, the region was under the British indirect rule administration.
4. The Southwest region is 25 410 km^2^ and its population was estimated at 1 853 000 inhabitants as of 2015. Of the 18 health districts of the region, four were implementing PBF.

The Southwest is an English-speaking region that was under the British indirect rule administration up until the early 1960s. The Southwest houses the only oil refinery in the country, along with important tea, cocoa and rubber plantations.

Of the 661 HFs implementing PBF throughout the four regions, 481 were in the intervention Group (129 in the East, 22 in the Northwest, 22 in the Southwest and 308 in the Littoral), and 180 were in the control Group (56 in the East, 64 in the Northwest and 60 in the Southwest).

As we wanted to deepen the mechanisms PBF put in place to facilitate the availability of EMs and how these mechanisms interacted with the overall context of the health system to produce or hinder the availability of EMs, we restricted our study to the intervention group.

In the pre-assessment phase, we categorized intervention facilities according to their levels of performance between 1 July 2012 (start of the programme) and 30 December 2014. The performance was calculated as the relative increase (T_r_) in the performance payment received between the start (P_i_) and the end of the randomization phase (P_f_) of the intervention, i.e. T_r_ = [(P_f_ – P_i_)/P_i_]×100. This categorization was further validated by key informants of the central level of the Ministry of Health (MoH). We selected contrasting HFs (low and high performance) and also took into consideration the location (urban vs rural) and the type (public vs private). Public HFs are directly supported by the Government and manage their expenses with Government budgets. Conversely, Private-for-profit and Confessional HFs manage their expenses from their own investment or self-generated income. Consequently, the costs of services are theoretically high in these two last categories of HFs compared with those of the public group. All three types of HFs provided the same basic package of activities, although some particularities existed. For example, private HFs were less keen to offer preventive services. As for the location of the HF (i.e. rural or urban), the main characteristics relied on their population catchment area, frequentation and geographical accessibility. HFs in rural areas tended to have less population and patients, and to be also less accessible.

Six HFs per region, for a total of 24 facilities in 15 health districts (four in East, four in Littoral, three in Northwest and four in Southwest), including 3 privates for profit, 3 confessional and 18 public HFs, were retained for the study. Among the 24 HFs, five were located in urban areas. These different locations, settings, as well as owners of the facilities, provided a range of relevant experiences to our subject of interest (see Table 1).

**Table 1:** HFs included in the study

No	Health facilities	Region	District	Performance	Owner	Location
1	Mindourou	East	Abong-Mbang	High	Public	Rural
2	Seguelendom	East	Doume	High	Public	Rural
3	Bika	East	Nguelemendouka	High	Public	Rural
4	Djaposten	East	Abong-Mbang	Poor	Public	Rural
5	Oundjiki	East	Kette	Poor	Public	Rural
6	Bayong	East	Doume	Poor	Public	Rural
7	Loum I	Littoral	Loum	High	Public	Urban
8	Charité	Littoral	Cité des Palmiers	High	Private for profit	Urban
9	Delangue	Littoral	Edéa	High	Public	Rural
10	Bikefo	Littoral	Cité des Palmiers	Poor	Private for profit	Urban
11	Espoir	Littoral	Loum	Poor	Private for profit	Urban
12	Bonepoupa	Littoral	Yabassi	Poor	Public	Rural
13	Mentang	Northwest	Fundong	High	Public	Rural
14	Mbontsem	Northwest	Kumbo-East	High	Confessional	Rural
15	Bamessing	Northwest	Ndop	High	Public	Rural
16	Ngendzen	Northwest	Kumbo-East	Poor	Public	Rural
17	Finkwi	Northwest	Ndop	Poor	Confessional	Rural
18	Bamukah	Northwest	Ndop	Poor	Public	Rural
19	Dikome	Southwest	Kumba	High	Confessional	Rural
20	Big Ngbandi	Southwest	Kumba	High	Public	Rural
21	Bova	Southwest	Buea	High	Public	Rural
22	Bakingili	Southwest	Limbe	Poor	Public	Rural
23	Kendem	Southwest	Manfe	Poor	Public	Rural
24	Limbe	Southwest	Limbe	Poor	Public	Urban

### Population sample and recruitment

We interviewed a total of 55 participants. The selection of participants was purposive. This approach is often used in qualitative research to identify actors who have some characteristics or are in situations pertinent to the object being studied (Gilson *et al.*, 2011). We sampled the three types of actors involved in the decision triangle (Monnier and Spenlehauer, 1992; Ridde and Diarra, 2009), selecting information-rich cases using job profiles, the main criteria being respondents’ knowledge of the PBF programme and the drug supply system.

Monnier and Spenlehaur (1992) describe three major roles that define the different actors of the decision triangle: the role of legitimation for qualified decision-makers, those who formalize decisions (i.e. the legitimizers), the role of action held by the implementers, those who operationalize those decisions (i.e. the actants), and finally that of the reaction to the policy envisaged or ongoing (i.e. the reactants). Legitimizers have an institutional power that does not stem solely from their status but also from their roles and capacities of integration between the different actors. They have no operational responsibility. In our study, the legitimizers included policy-makers of the central and regional levels of the MoH. Actants are actors who have views that in theory reflect the mandate they hold; however, the mandates are very often defined in an imprecise way, leaving them important room to manoeuvre. These actors included health workers, private wholesalers, and technical and financial partners of the PPA. Reactants mainly include the disparate set of civil society actors subject to or beneficiaries of the policy (i.e. users of HFs and members of the community) (Table 2).

**Table 2:** Summary of respondents by type, level and number

Actors	Level of work	Work profile (region/district)	Role in the decision triangle	Number of informants interviewed	Description of data collected and topics covered
Policy–makers	International	Technical and financial partners	Legitimizers	1	Institutionalization of PBF PBF and drug supply system liberalization Quality control of EMs
	Central—MoH	National co-ordinator of the PBF programme Director of the co-operation unit National PBF technical expert	Legitimizers	3	Institutionalization of PBF PBF and facility autonomy PBF and drug supply system liberalization Factors facilitating or impeding changes in access to EM Quality control of EMs Enforcing regulation
	Central—Public supply drug system for EMs	Director and deputy director of CENAME	Legitimizers	2	PBF and facility autonomy PBF and drug supply system liberalization Quality control of EMs Implementation challenges and solutions developed
	Regional—Delegation of public health	Regional Delegate of public health (East, Littoral, Northwest and Southwest)	Legitimizers	4	Institutionalization of PBF PBF and facility autonomy PBF and drug supply system liberalization Factors facilitating or impeding changes in access to EM Quality control of EMs Enforcing regulation
Implementers	National	Private wholesalers	Actants	2	PBF and drug supply system liberalization Quality control of EMs
	Regional	Managers of the PPA (East, Littoral, Northwest and Southwest)	Actants	4	PBF and facility autonomy PBF and drug supply system liberalization Implementation challenges and solutions developed PBF and EMs availability Factors facilitating or impeding changes in access to EM Enforcing regulation
	Regional	Managers of the RFHP in all the four regions (East, Littoral, Northwest and Southwest)	Actants	4	PBF and facility autonomy PBF and drug supply system liberalization Quality control of EMs Implementation challenges and solutions developed
	Regional	Private wholesalers	Actants	4	PBF and drug supply system liberalization Quality control of EMs
	Peripheral	District medical officer (Doume, Fundong, Loum, Ndop and Manfe) Chief of HC (Abong-Mbang, Doume, Kette, Cité des Palmiers, Fundong, Kumbo-East, Kumba, Ndop, Limbe and Buea) Pharmacy attendant (Yabassi, Limbe, Nguelemendouka, Ndop and Edéa)	Actants	5 11 5	PBF and facility autonomy PBF and drug supply system liberalization Implementation challenges and solutions developed PBF and EMs availability Factors facilitating or impeding changes in access to EM Enforcing regulation
Beneficiaries	Community level	Community-based organizations and health committee representatives (Kette, Cité des Palmiers, Ndop, Kumba, Doume, Edéa, Fundong, Buea, Yabassi and Manfe)	Reactants	10	PBF and access to care PBF and care-seeking pathways PBF and EMs availability
Total				55	

CBO, community-based organizations; HCo, health committee; PBF, performance-based financing; EM, essential medicine; RFHP, Regional Funds for Health Promotion; CENAME, national essential drugs and disposables procurement centre; MoH, Ministry of Health.

As a first step, during a meeting organized by the MoH in March 2015 in Yaoundé, we conducted interviews with four regional level PBF managers and the national coordinator of the PBF programme, who played a role in formulating and implementing the PBF programme. In interviewing these key stakeholders, we also asked them about prospective participants to reduce selection bias. Each prospective participant was contacted by phone either by the first author or by a trained research assistant. Between April 2015 and May 2015, we conducted interviews with 50 participants, including 2 policy-makers at the central level, 21 health workers, 6 actors in the public supply drug system, 6 private wholesalers, 1 actor from international organization and 10 members of community-based organizations and health committees (see Table 2 for more details). The participants did not receive any incentive nor compensation in exchange for their participation in this study.

### Data collection

Data were collected through in-depth interviews (*n* = 55). In the qualitative approach, interviews are frequently seen as one of the best techniques to ascertain the other person’s perspective (Patton, 2002, p. 341; Puebla *et al.*, 2004). Information was collected using semi-structured questionnaires. A semi-structured guide is useful when gathering information from key informants who have personal experiences, attitudes, perceptions and beliefs related to the topic of interest (Kallio *et al.*, 2016). It can help to explore new concepts and generate hypotheses (DeJonckheere and Vaughn, 2019). The interview guides were developed based on the study objectives and informed by the content analysis (Hsieh and Shannon, 2005) of some key policy documents (AEDES, 2012; Ministère de la Santé Publique, 2012; Le Mentec and Mettling, 2014). To minimize any interference in the daily participants’ work, interviews were conducted face to face (*n* = 47) in an interviewee’s chosen place and time or via videoconference (*n* = 8) by the first author and trained research assistants and lasted about 40 min each. The interviews were conducted in English (*n* = 32) and French (*n* = 23), audio-recorded and supplemented by note-taking. These interviews aimed to elicit the emic perspective (Kottak, 2015) on (1) the factors that facilitated or impeded changes in access to EM (supervision, coaching, governance, leadership, accountability, knowledge, attitude, practice, …); (2) the drug procurement system; and (3) the effect of the PBF intervention on EMs availability (stock-out of EMs, patient satisfaction), and part of the questions were linked to the theory of intervention. Different themes were used for different participants. For policy-makers, the interview focused on the institutionalization of PBF, PBF and the liberalization of the drug supply system, the quality control of EMs, and the enforcement of the regulation. Interviews with health workers targeted the autonomy of HFs under PBF, implementation challenges and solutions developed, and factors facilitating or impeding changes in access to EM. Beneficiaries were asked to give their view on the availability of EMs in the PBF context (Table 2). All interviews were recorded and transcribed verbatim in the primary language (English or French) by trained research assistants.

It should be noted that we conducted our data collection (March to May 2015) before the IE was over (April to June 2015) and hence our study was not a follow-up to the quantitative IE.

### Data management and analysis

Transcripts were recorded verbatim and entered into QDA Miner Lite (Provalis Research, Montreal) for analysis. Each transcript had a unique identifier comprising date, region code, participant type, level of work, HF’s code, allowing exploration of data by subgroup status (e.g. HF performance). Analysis followed the four practical stages suggested by Marshall and Rossman (2006) of ‘organizing the data’, ‘generating categories, themes, and patterns’, ‘testing any emergent hypotheses’ and ‘searching for alternative explanations’.

Coding into themes was carried out in a 2-fold manner. The second author developed the initial coding scheme, based on the literature and on the themes included in the interview guide, then discussed it with the research team and came to an agreement on the coding and categorization. The second phase of coding was during transcription, where prominent issues were marked and further discussed within the research team.

Two researchers independently coded the transcripts of all the interviews and field notes using the agreed coding and categorization. We applied the predefined codes to the transcribed material, but allowed new codes to emerge where coding categories were directly derived from the text data (Hsieh and Shannon, 2005) as we proceeded through reading. The lead author, in a more deductive approach, drew on thematic analysis strategies (Miles and Huberman, 1994; Corbin and Strauss, 2015) to look for patterns, specifically grouping codes into named patterns of ‘respondents’ perception on the availability of EMs’, ‘causal pathways that influenced the availability of EMs’ and ‘mediating factors affecting the ability of the programme to produce expected changes’, as well as ‘concerns about the PBF intervention in relation to the supply system of EMs’. These patterns were then used to structure and summarize the data, and to ultimately build overall findings.

To ensure the credibility of this study, we started data analysis in the field, forming an iterative relationship with key policy document analysis and interviews (Maxwell, 2005). We applied analytic triangulation (Patton, 2002), whereby data were coded independently by the first author and one research assistant. We used the opportunity of a national PBF meeting that held in June 2016 to discuss our preliminary findings with selected key informants from the central (*n* = 3) and regional (*n* = 4) levels of the MoH, allowing us to validate or refine our final interpretation (Miles and Huberman, 1994).

### Ethics approval and consent to participate

The study protocol was reviewed and approved by the Cameroon National Ethics Committee for Human Health Research (N0 2015/02/549/CE/CNERSH/SP) and the WHO Research Ethics Review Committee (RBF2014–395). Administrative authorization was granted by the Cameroonian Ministry of Health (D30-298 L/MINSANTE/SG/DROS/CRSPE/CEA2) prior to data collection. All respondents provided verbal or written informed consent before interviews. To ensure the confidentiality and anonymity of the data, we replaced participants’ names with codes.

## Results

It was clear for almost all interviewees that drug availability was a challenge that existed before PBF. The choices of drugs in the pharmacies were limited and affected by frequent stock-outs. Patients sometimes had to travel far to buy drugs prescribed in facilities. According to respondents, the PBF intervention had positive effects on the availability and variety of drugs. Respondents reported that these effects progressively minimized stock-outs. Key findings are displayed to (1) present the causal pathways that influenced the availability of EMs and present the possible mediating factors affecting the ability of the programme to produce expected changes, and (2) highlight concerns about the PBF intervention in relation to the supply system of EMs. In addition, findings are also presented to highlight differences between high- and low-performing HFs (Table 3).

**Table 3:** Summary of characteristics of high- and low-performing HFs

	High-performing HFs	Low-performing HFs
Liberalization	Good understanding of the principle of liberalization List of accredited suppliers available	Poor understanding of the principle of liberalization due in part to communication gap No formal text to explain the principle of liberalization
Autonomy	Managers recognize pharmacy as the HF’s property Hiring of extra pharmacy attendant to enable shifts for 24/7 services Managers buy drugs based on the health centre needs Managers use HF income to do some repairs in the pharmacy as well as buy pharmacy equipment	Not always clear for managers if the pharmacy is fully under their control Managers wait for the RFHP to supply EMs
Enforcing regulation between DMTs and HFs	Similar pattern concerning the frequency and the intensity of quality evaluation, supervision and inspection.	
Greater accountability of the pharmacy attendant and transparency of the pharmacy management	More frequent internal supervisions by the HF manager Onsite capacity building of the pharmacy attendant Improved pharmacy attendants’ perceived ability to do their job Improved responsiveness of the pharmacy attendant Pharmacy attendants are proud and confident as they are feeling valued	Few or no internal supervision No or very low capacity building plan for the pharmacy attendant
Contextual factors	Good access to catchment area Two or more pharmacy attendants HFs well equipped	Bad access to catchment area One or no pharmacy attendant Small size population of the catchment area
Leadership and management	Strong performance management of the pharmacy with key indicators tracked and displayed on a wall, Monthly meetings where the pharmacy performance is presented and strategies to improve discussed Consensus-based decisions with clear responsibilities, and followed up for implementation Strong leadership of the manager concerning the pharmacy management.	Little process for planning for pharmacy activities, and performance tracking Manager of the health centre often not available Irregular meetings with staff and the pharmacy performance not often discussed
Unintended consequences	Internal mobilization of funds (loans and savings) Participation of the staff in the capital of the pharmacy, with shares producing profits	Fragmentation of the drugs management system whereby pharmacy attendants carefully manage only ‘PBF drugs’

### Pathways that influence the availability of EMs

**Figure 2:**
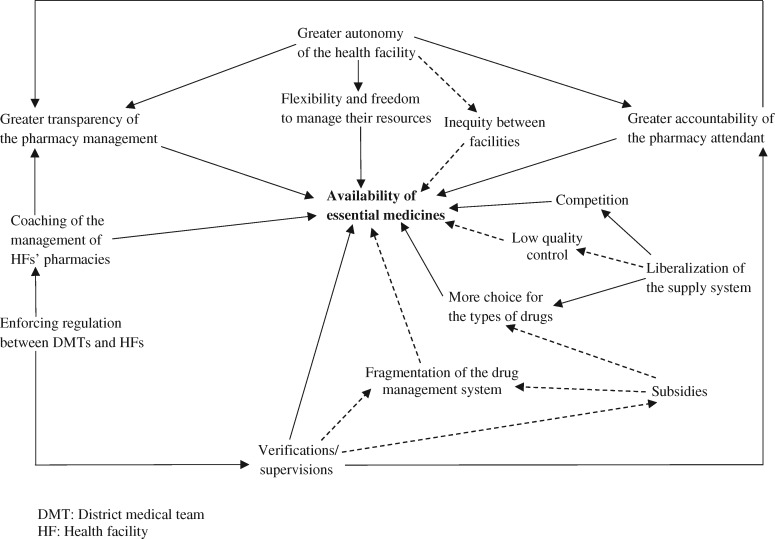
Pathways by which PBF can influence the availability of EMs. DMT, district medical team; EMs, essential medicines; HF, health facility.

The findings highlight the possible pathways through which the PBF intervention influenced the availability of EMs in facilities. More specifically, the results suggest that the availability of EMs was linked to supply system liberalization, the increased autonomy of facilities, the enforced regulation from the district medical team (DMT) and the greater accountability of pharmacy attendants. Figure 2 presents some of these links. The availability of EMs induced by PBF according to stakeholders’ views is at the centre, surrounded by several elements that PBF put in place or focused on. These elements are linked to each other using arrows. The direction of arrows indicates that a change in the first element influenced the second element, which ultimately led to changes in the availability of EMs. Broken lines highlight elements raising concerns.

#### Liberalization of the supply system

The PBF intervention changed the existing institutional configuration by bringing new actors into the healthcare system. More specifically, the principle of liberalization led to the introduction of new accredited actors in the provision of drugs. Key stakeholders explained that opening the public national EMs supply system in Cameroon market to the private sector was one of the noticeable actions of the programme. As one informant put it:

*In fact, we go to ‘the lowest bid’. It's business […] L-PHARMA does not offer generics. Only specialties. […] But those ones, we get them from P-PHARMA, they are cheaper to buy, for the same quality* (Manager health facility, Peripheral level, Loum health district).

Respondents believed that the liberalization brought changes such as the possibility of having more drugs available as well as having more choices of drugs at the wholesalers’ level. However, the influence of the liberalization of drug prices remained limited. Respondents said that most private wholesalers were subject to heavy taxes that the national essential drugs and disposables procurement centre did not have because the latter benefited from numerous fiscal advantages. Consequently, PBF did not considerably influence the prices of drugs sold by private wholesalers.

There were noticeable differences between the participants’ understanding of the principle of liberalization. Managers of facilities who understood the principle of liberalization seemed to be remarkably motivated and were keener to do more to improve the performance of their pharmacy compared with those with a lesser understanding of the concept. It also appeared that managers of top performing facilities had good understanding of the principle of liberalization compared with health workers in poorer performing facilities. These two respondents in top- and poor-performing facilities illustrate this:

*Liberalization pertains to our freedom and rights to buy our essential medicines where there are advantages for us, in term for example of price and type of the product. You will notice that our pharmacy has changed a lot since we have the possibility to buy essential medicines from various suppliers* (Manager health facility, Peripheral level, Doume health district).

*Euh… I once heard from a colleague in a nearby facility that the ministry will put in place many organisations to supply facilities with EMs because the RFHP that is currently doing the job is overwhelmed and not performing well. That said, we are still buying our drugs only from the RFHP and it is what we were doing before that we are doing now* (Manager health facility, Peripheral level, Ndop health district).

There were diverse views about the reasons for the poor understanding of the liberalization in poor-performing facilities. Although some facility managers pointed out the communication gap between the district and facilities, others highlighted their limited comprehension of their power over the pharmacy management.

Although managers of the poor-performing facilities felt that they were informed about the EM supply system liberalization, they pointed out inadequate communication in the planning and implementation phase as a source of much frustration, as highlighted by the following quote:

*In the launching meeting in Bamenda, we were told that over the course of the program, we will be granted the authorization to buy drugs from other suppliers. However, things were not so clear. We did not receive any formal text nor directives to do so. After a year of implementation, we were informed during a coordination meeting that it is up to everyone to implement the mechanism. You see, they decided and just want us to act without any legal text signed* (Manager health facility, Peripheral level, Limbe health district).

All managers of the poor performing facilities said their workload increased significantly with the PBF programme and that this increase seriously compromised their daily practice and their management capacity. They expressed their view that the personal and professional impact of the PBF programme was negative. About two-thirds of them disagreed with the statement that ‘liberalizing the EM supply system increases the pharmacy income and the personal financial incentive’. In expressing negative views, the health personnel of these facilities appeared to draw a valuable difference between policy objectives and its implementation. For example, they pointed out the lack of support strategies to optimize and to standardize implementation with the aim that the intervention is as uniform as possible.

Respondents from the DMT further described how managers of the poor-performing facilities were not keen to move out of their comfort zone as they continued to manage their facilities without any change in their practices, as shown by the following extract, raising therefore questions regarding the quality of the supervision from the district:

*Since the beginning of the program, we have noticed that some chiefs of health facilities don’t want to change. We have explained to everyone that the essential drugs supply system is now open to many accredited providers, but health facilities that you observe with poor performance have still not changed their practices despite multiple supervisions* (Member of District Medical Team, District level, Kumbo-East health district).

When asked about what role they played in the implementation of the drug supply system liberalization, facility managers reported several strategies they put in place. Their opinion was that these strategies helped to improve the performance of their pharmacy. In particular, these strategies (e.g. regular staff trainings and meetings to discuss the drug supply system including liberalization, establishing a list of accredited EMs suppliers with the support from the DMT, mobilizing loans and savings) were only experienced in high-performing facilities. For example, two facility managers claimed that they leveraged loans with low interest among their personnel to buy the initial stock of drugs after the withdrawal of the RFHP from the pharmacy management. The following excerpt is from one of these participants:

*When we started to manage the pharmacy, we did not have funds to buy drugs. During a monthly meeting, one of our colleagues raised the idea that we could use our PBF incentives and put it as investment shares, so each month we could receive investment return as profit. We did it and for the past four months that we started it, we have been receiving at least 15, 000 FCFA [30 $US] per month, depending on the initial amount of investment. In addition, our pharmacy has now enough drugs, that is a win-win business* (Manager health facility, Peripheral level, Nguelemendouka health district).

More broadly, respondents suggested that without local support from the personnel, they would not be able to deal with the EM liberalization, pointing to the likely role of staff cohesion as a resource for implementation.

#### The autonomy of HFs

Before PBF, the centralized management system did not recognize any form of autonomy in the management of pharmacies in facilities. Staff at the operational level had no access to EMs revenues. Although these actors were previously considered to be executors in the distribution chain, PBF made them partners. The status of simple executors in a situation where actors felt that the benefit was far below their expectations favoured illegal practices such as the embezzlement of drugs and funds, insofar as actors resorted to parallel circuits other than the RFHP. Interviews with front-line health workers provided more insight:

*Public facilities are forbidden to get supplied elsewhere when the RFHP does not have all the drugs. It creates a black market. […]* (Manager health facility, Peripheral level, Cité des Palmiers health district).

Under the monopolistic regime, the distribution of EMs was described as a ‘push system’ associated with a central agency (RFHP) supplying facilities (Le Mentec and Mettling, 2014). Facilities used to wait passively for drugs and undertook little effort in the event of a stock-out. The RFHP determined the types and quantities of EMs to be distributed in facilities. Facilities were expected to provide stock and consumption information to the RFHP to assist planning. Field missions by the RFHP to supply as many facilities as possible were often carried out according to a planned itinerary, in order to minimize the costs of the mission (Le Mentec and Mettling, 2014). Thus, a facility experiencing a stock-out in-between these supply missions was likely to have no alternative solution.

Several managers of facilities felt that this situation was worsened by the fact that pharmacies were out of their control. With the liberalization, the new configuration of the distribution system put facilities in the vanguard. They became responsible for evaluating their needs and became also more responsive to stocking up their pharmacy in case of drug shortages. The new approach was seen as a ‘pull system’ coupled with liberalization, where facilities directly purchased drugs from accredited sellers. One informant explained:

*Our platform is the monthly meeting during which we evaluate our needs and also assess the previous month. During the month, if the facility faces a problem with drugs availability, the chief has the power to find solutions and then report it to the committee later on during the monthly meeting* (Manager health facility, Peripheral level, Fundong health district).

According to facility managers, the autonomy gave them the flexibility and freedom to manage their own resources (human, financial, equipment and drugs). Similarly, several facility managers mentioned having full control over the management of the benefits generated by the sale of medicines. Informants explained how surplus resources allowed them to improve the storage space and the working environment:

*We are now making considerable profit from the drugs and we are able to use it to construct more storage space. […]* (Manager health facility, Peripheral level, Buea health district).

Autonomy seemed to be much appreciated even by other members of staff as well as members of health committees at the community level. According to them, it had become easier to react quickly to any stock-out problems. From their perspective, it was really when facilities were granted more autonomy that the shortage of drugs decreased, as one community member expressed:

*To speak frankly, the issue of stock-out was very common in our facility. After one year of the program implementation, our facility started to be completely autonomous. From that period, there is no more shortage of drugs in our facility* (Member of health committee, Community level, Manfe health district).

However, results showed that the principle of autonomy stood against the interests of certain national essential drugs and disposables procurement centre and RFHP actors, in the sense that it might lead them to lose hidden advantages such as baksheesh. The preservation of their interests is an element that weighed a lot in their behaviour when implementing the principle of autonomy. One informant recalled:

*You know it’s not everyone who will agree with the autonomy principle. For some, it makes a kind of hollow in the pocket if they don’t sell their drugs* (Manager health facility, Peripheral level, Edea health district).

Although the advantages of autonomy were clear for most actors at the operational level, it faced an organizational culture of procedures and inaction that characterize public organizations in Cameroon (Ngouadjio, 2014). This led to a clash between the application of the autonomy principle and the defense of self-interests of certain actors. HFs, freeing themselves from the yoke of the RFHP, seemed to enjoy a greater autonomy and readability in the management procedures. This was noted by the key informants:

*In the PBF when you are in charge, you manage all your resources including drugs. So, the facility decides or not where to go for drugs, and is no more accountable to RFHP. […] The chief of the facility manages with his staff. [It’s]not that you sell and somebody in the regional city tells you that it is up to the RFHP to manage your drugs* (Manager health facility, Peripheral level, Loum health district).

The views and experiences of participants regarding autonomy varied across facilities with noticeable differences between health workers from good- and poor-performing facilities. For example, facilities that autonomously recruited and managed their pharmacy attendants acknowledged the highest performance in the three regions, whilst managers from low performing facilities tended to believe that autonomy would never be a reality because it is very complex to manage. Some facility managers from high- and low-performing facilities recalled:

*Autonomy is really a good thing. We have recruited additional staff for the pharmacy, and this is something that would has never happened without PBF* (Manager health facility, Peripheral level, Ndop health district).

*The image that people have outside is that we are not doing anything, yet to us, it’s not deliberate, it’s just that the business is very complex. We have been trained on how to manage our resources, though it is not easy for us to apply everything* (Manager health facility, Peripheral level, Kette health district).

At the same time, the vast majority of respondents made it clear that the autonomy process remained partial. They had concerns regarding the ability and willingness of the RFHPs to fully transfer the management of pharmacies to facilities. From their point of view, some RFHP actors even attempted to block the process, refusing e.g. to carry-out the initial inventory of the pharmacies of health centres, a preliminary step toward the autonomy.

#### Enforcing regulation between DMTs and HFs

The most outstanding changes experienced by interviewees regarding external supervision of the pharmacy were its regularity and intensity. Most respondents perceived an increase in the availability of EMs in their facility as a consequence of more supervisions and coaching from the DMT. DMT was reported to visit facilities on a monthly basis and used a structured supervisory checklist. In addition, on a quarterly basis, they assessed structural quality components (i.e. physical and organizational elements of healthcare) and treatment protocols. Given that the scores that pharmacies received influenced bonus amounts, pharmacy attendants and facility managers considered this process to be vital to their achievement in the programme. They felt continuously inspected but were happy about the supportive nature of the enhanced supervision and coaching. The improved performance resulted from close formative supervision, coupled to the projection of earning more money, as one informant expressed:

*The DMT comes here at least once a month for the supervision and each time, they spend half day with the chief and me to review the management of the pharmacy. When they notice a problem, they tell us how to do and to avoid it in the future* (Pharmacy attendant, Peripheral level, Fundong health district).

The monitoring and evaluation indicators that were included in the quarterly contract and business plan, and that contained indicators regarding EMs, were used to assess the achievement of the facilities’ objectives. In case of a breach of contract, facilities incurred sanctions ranging from temporal to permanent suspension of the contract. A member from the community explained:

*Our health centre had been suspended for three months. […], well … I was told that something was wrong with the management of the pharmacy. […]* (Member of a CBO, Community level, Abong-Mbang health district).

When we asked the district health authorities what they thought about their role in influencing the availability of EMs, they responded that they considered themselves to be at the centre of the intervention. They received funding from the PBF programme to improve their supervisory and regulatory roles. They said that in their own contract with the PPA, they were evaluated based on the number of formative supervisions and on the coaching of the management of pharmacies. In addition, payments to the DMTs were adjusted based on the availability of tracer drugs, the storage of EMs stocks, the absence of expired drugs and the financial management related to the sale of EMs. They also insisted on their mandate to enforce the utilization of protocols and guidelines by health workers to improve rational prescription in facilities as well as their mandate to penalize facilities that could not meet policy norms.

Comparisons between participants from high- and poor-performing facilities regarding the regularity and intensity of the supervision, as well as its content, revealed similar patterns.

Although the majority of respondents described positive changes in enforcing the regulation, many of them also mentioned how implementation-related challenges attenuated these changes. These challenges were linked to human and financial constraints. The most cited example was the delays in payment from the PPA, leading to the demotivation of the DMTs. They reported that in such situation, they had to cancel supervision missions or just carry it out very superficially.

#### Greater accountability of pharmacy attendants and transparency of pharmacy management

Before PBF, pharmacies were extensions of the RFHPs, with no direct link with the facilities’ management teams. With the advent of PBF’s principle of autonomy, this aspect was changed, and pharmacies became the property of their corresponding facility. Pharmacy attendants became supervised at the local level. Moreover, for almost all the cases, the pharmacy attendants were recruited locally and were not civil servants. They could be fired for not achieving objectives. A respondent stressed this, saying:

*Last year our first pharmacy attendant was fired after only six months of work. […]* (Manager health facility, Peripheral level, Kumba health district).

However, some pharmacy attendants expressed concern over their fragile position as some facility managers could abuse their power. One respondent said:

*Everyday, I am just afraid that the manager [chief of the facility] can end my contract overnight. He once told me that he is the boss and that he can even stop my contract without any reason* (Pharmacy attendant, Peripheral level, Ndop health district).

In general, all pharmacy attendants supported the idea that PBF had made them more professional in their duty. First, they acknowledged that they benefited from onsite trainings and supportive supervisions from DMTs allowing them to improve their skill on stocks management, reporting and planning. These trainings were key to identify priorities in service and areas in need of improvement, as well as developing solutions. Secondly, PBF brought more clarity regarding their duties by elucidating their term of references and explicitly linking it to their performance salary. Finally, about a third of pharmacy attendants believed that they had been given more responsibilities, making them proud and confident.

Both implementers and policy-makers highlighted the perceived improvement of transparency of the pharmacy management. Facility managers became more proactive in reporting the pharmacy’s financial accounts. Numerous intervention practices were important to this effect, including the continuous performance monitoring and feedback, coupled with quality evaluation, enhanced supervision and coaching by both the PPA and the DMT. A respondent stressed this by saying:

*The implementation of the business plan states how we should report our financial accounting, be it the one of the pharmacy, the laboratory, or any other service. Each month, we display our financial statement and the product of the indice tool on the wall […]* (Manager health facility, Peripheral level, Nguelemendouka health district).

In high-performing facilities, PBF improved the pharmacy attendants’ perceived ability to do their job. Pharmacy attendants also voiced their appreciation for how the programme helped them develop their skills. They reported how the improved managerial environment resulting from the intervention made them feel more effective in translating their skills into practice and become more accountable. As one pharmacy attendant stated:

*Let me take an example. With the PBF program, we established monthly coordination meetings during which each unit must present detailed monthly managerial strategies, activities, statement of accounts and perspectives of the months forward. This is how we are on a continuous learning process and implementing what we are learning. I can assure you that I am now feeling more comfortable doing my job* (Pharmacy attendant, Peripheral level, Kumbo-East health district).

Many health workers further described how they felt that their work teams had improved service delivery by lowering drug shortages and increasing responsiveness.

In low-performing facilities, however, perceptions about the improvement of the transparency of the pharmacy management remained much more mixed. Although respondents generally agreed that the intervention could improve the management of the pharmacy, they were not able to support their statement with concrete facts.

### The intervention also generated concerns

#### Worsening of the quality control of EMs with the liberalization of the drug supply system

The regional health delegation’s mandate of regulation included drug quality control. Private wholesalers had to get accreditation before any drug was supplied to facilities. However, this accreditation was likely based only on administrative elements and not technical ones (testing the authenticity of the drugs, their active ingredients, the dosage, the composition, the weight, etc.). In fact, the regional delegations in charge of giving these accreditations did not have the expertise to conduct quality control. Without a real quality control mechanism at the regional level, many of the interviewed actors expressed their worries concerning the deterioration of drug quality. A respondent who played a key role in implementing the programme gave us more insight:

*I must acknowledge that our quality control remains very poor. The quality of a drug is not who the manufacturer is but how it is handled. […]* (Member of regional delegation of public health, Regional level, Littoral region).

According to regional stakeholders, monitoring and quality control should be facilitated and reinforced because of the various sources of drugs.

#### Concerns that the autonomization of HFs leads to inequalities

According to informants, the principle of solidarity was an advantage of the supply system implemented prior to PBF. The drug supply system management was centralized at the level of the RFHP, which had the mandate to supply all public HFs, regardless of their size and location, through a push system. For example, respondents explained that during supervision missions prior to PBF, the RFHP picked out drugs with a close expiry date in one facility and repositioned them in another facility that had huge volume of activities or shortages of these drugs. Risks and administrative costs were, therefore, shared between facilities, as pointed out in one interview:

*It is because of the high income of the people in the urban area that we can sustain the rural area pharmacy. Actually, we've calculated that a pharmacy that does not make sales of >2 million CFA francs [$4 000 USA] a year cannot comfortably pay a pharmacy attendant, pay the transportation, […]. So, if the solidarity system was to stop, then I think those pharmacies will not be sustainable* (Member of the Regional Fund for Health Promotion, Regional level, Northwest region).

Participants argued that with the application of the PBF’s principle of autonomy, small facilities that were not very active or that were hard to reach experienced financial difficulties and more drug stock-outs. These facilities faced low utilization and consequently low-cost recovery, especially for EMs. With PBF, these facilities started to buy small quantities of drugs on a regular basis because they did not have enough funds to acquire large quantities as they used to with the RFHP. As a result, fixed costs exploded, and funds to restock the pharmacies dwindled, leaving room for frequent stock-outs. The low resource mobilization faced by small facilities and those hard to reach was not only challenging for the facilities’ budgets, and subsequently the work environments but also for the pharmacy attendants’ enthusiasm to be present. One informant explained:

*Incentives are good. Unfortunately, we do not have them […]. They are good because they improve on one’s motivation. Let me give you an example of the maternity ward. For the last three months, we have received less than ten deliveries, so our recovery cost is very small and so does our PBF income. At the end of the day, our revenue is not enough to replenish our pharmacy stock and to incentive the staff* (Manager health facility, Peripheral level, Kette health district).

#### Fragmentation of the drug management system

One of the PBF programme’s objectives was to improve the health information system (AEDES, 2012), including that of the EMs supply system. In this respect, the PBF intervention did not advocate for the use of additional forms, registers or tools for data management. However, actors in the field sometimes were tremendously creative (in the ironic sense), focusing more on strategies that improved their personal gains in the programme, such as the development of parallel drug management systems to improve only the indicators paid for by the programme. A respondent stressed this, saying:

*During our supervision in the field, some facilities were using separate drug cupboards for the ‘PBF drugs’ […]. There are some specific tracer drugs that the PBF qualitative evaluation checks. Facilities tend to put more emphasis on those tracer drugs* (Member of district medical team, District level, Limbe health district).

## Discussion

This study makes a unique contribution to the literature by exploring how a PBF intervention can influence the availability of EMs at the primary healthcare level. The Cameroon PBF case placed the drug supply system at the centre of its design, with the RFHP identified to take over the performance purchasing role from the early stage of the programme in order to facilitate its sustainability and ownership in the long term (Sieleunou *et al.*, 2017).

Our findings, though cautious in the absence of more evidence, suggest that the PBF programme improved the perceived availability of EMs in three regions in Cameroon.

Many studies have observed an association of the PBF programmes with significant improvements in availability and reductions in stock-outs of medicines and medical supplies in Sub-Saharan Africa (Canavan and Swai, 2008; Vergeer, 2008; Soeters *et al.*, 2011; Binyaruka and Borghi, 2017; Rudasingwa and Uwizeye, 2017). In contrast, findings from Afghanistan (Engineer *et al.*, 2016) and Burundi (Bonfrer *et al.*, 2014; Rudasingwa *et al.*, 2015) did not evidence any impact of PBF on drugs availability. These mixed findings highlight the fact that the effects of the PBF programmes on the improvement of the EMs availability may vary depending on the intrinsic design of the programme and the specific characteristics of the context (Witter *et al.*, 2013; Ogundeji *et al.*, 2016; Bertone *et al.*, 2018; De Allegri *et al.*, 2018). This is also in line with emerging evidence suggesting that PBF is likely to work differently depending on initial performance levels (De Allegri *et al.*, 2018).

Though the benefit of the intervention theory was that it offered an opportunity to synthesize several concepts related to the PBF programme and guide the data collection and analysis, we went beyond it to look at some consequences that were not directly targeted by the intervention. We identified four major pathways through which the PBF intervention can influence the availability of EMs in facilities in Cameroon. These include the autonomy of facilities, supply system liberalization, the enforced regulation from the DMT and the transparency of the pharmacy management and greater accountability of the pharmacy attendants.

Autonomy over human and financial resources has been described as a *sine qua non* for governing and increasing the performance of public hospitals (De Geyndt, 2017; Mayumana *et al.*, 2017). Yet, researchers described stakeholders at the central level as devisors of strategies to maintain their power and to limit the facilities’ autonomy (De Geyndt, 2017). In Cameroon, facilities had autonomy that could be leveraged to impact the supply system, and this appeared as a catalyst for more EMs availability. However, the autonomization process in Cameroon did not go far enough and ‘autonomy existed in theory but in practice it was limited’ (De Allegri *et al.*, 2018, p. 4), keeping facilities from reaching true improvement of EMs availability.

Few PBF programmes have proposed alternatives to the state monopoly on EMs supply. Therefore, Cameroon’s experience offers a valuable insight as it suggests that in the context where the state monopoly failed (Ngouadjio, 2014), liberalization of the EMs supply system could improve their availability. Nevertheless, this study also highlights the possibility that the liberalization of the drug supply system could worsen pre-existing inequalities between facilities. The failure in improving equity in hard to reach areas mirrors previous studies (Friedman and Scheffler, 2016; Ridde *et al.*, 2018). Therefore, policy-makers are called upon to implement suitable interventions to ensure equity throughout the country, such as supporting drug replenishment and transportation cost (as equity bonuses) for facilities in hard to reach areas (De Allegri *et al.*, 2018).

Supply chain quality control and assurance in most low- and middle-income countries are still poorly understood. Yet it is also documented that poor quality of drugs is found or reported in those countries (Giralt *et al.*, 2017; Van Assche *et al.*, 2018). Our results revealed concerns among stakeholders about the quality of EMs in the context of opening the supply system to private wholesalers.

In this context, any viable solution that improve drug quality will include strengthening the drug regulatory system, decentralizing the inspectorate at the regional level, enforcing quality standards and licensing in accordance with international standards.

Our data show that stakeholders in Cameroon perceive PBF as an approach to improve the accountability of pharmacy attendants through skill improvement, duty clarification and performance-oriented salary. Past research found that healthcare providers who are aware of their duties and responsibilities, as advocated by the PBF best practices, are more likely to better perform their work and improve the quality of care (Manongi *et al.*, 2006). Contextual differences appeared in the literature, indicating on the one hand the limitations of a PBF programme to overcome external influences on the behaviour of healthcare providers and, on the other hand, the complexity of the relationship that may exist between supervisory activities and their influence on the behaviour of healthcare providers. This is an indication that differences in the relative importance given to specific aspects of PBF design also produce differences in outcomes (Bertone *et al.*, 2018).

Our results highlighted the central role of the HF manager in optimizing the identified pathways which may, at least partially, lead to the optimal availability of EMs. The work of health workers at the operational level can be hard and challenging, as they habitually have to deal with working under excessive pressure resulting both from patients’ and hierarchy’s expectations. As first coined by Lipsky in 1980, the ‘street-level bureaucracy’ theory says that a successful implementation of public policy is not solely determined by the quality of policy measures, but instead is for a large part dependent on the actions of those who implement it, the front-line workers (Lipsky, 2010). These actions can lead street-level bureaucrats to take either a supportive or unsupportive attitude towards policy initiatives (Erasmus, 2014). This was evidenced in our study by some coping strategies, such as mobilizing loans and savings to buy the initial stock of drugs, developed by HFs managers to improve their performance. The findings of this study extend the available evidence on how street-level implementers control the complexity of day-to-day work over the course of a policy change, as seen e.g. in Burkina Faso, where health workers managers expressed creative strategies to promote facility deliveries (Belaid and Ridde, 2015), and in South-Africa, where front-line health workers adopted coping mechanisms to face the frustrations of their working environments (Walker and Gilson, 2004).

An acknowledged concern from stakeholders has been the fragmentation of the drugs management system whereby pharmacy attendants carefully manage only ‘PBF drugs’ (i.e. tracer drugs that are assessed with the qualitative checklist). The findings of this study confirm other studies’ evidence about unintended consequences that a programme such as PBF can cause beyond the targeted objectives of the intervention (Turcotte-Tremblay *et al.*, 2017). On the other hand, this also supports previous research indicating that if not carefully designed and implemented, a PBF programme could lead to system fragmentation (Canavan and Swai, 2008; Kalk *et al.*, 2010). Therefore, PBF intervention should be part of a more comprehensive and coherent approach to strengthen the whole health system (Meessen *et al.*, 2011; Witter *et al.*, 2019).

### Study limitations

It is important to emphasize that our findings represent mostly the experiences of actors involved in PBF. Given that most of them wished the programme to last despite the various challenges (Sieleunou *et al.*, 2017), and were therefore likely interested in portraying the intervention positively, it should be noted that relying on these actors’ self-reporting and social desirability could elicit some bias in interpreting the results. Rigorous quantitative methods and long-term observation in a real-life setting can be used to further develop and test our findings on how PBF might affect the availability of EMs. Moreover, the autonomization process of facilities as well as the liberalization of the EMs supply system were still ongoing at the time of data collection, and some elements that emerged later may not have been seized. A follow-up study would be useful to offer more understanding on how the facility autonomy and liberalization of EMs supply system affect EMs availability at the primary healthcare level over time.

In conducting our interviews, we used different topic guides for different participants. Moreover, the data collection strategies involved several interviewers, and both in person and by telephone approaches. These may have increased the variation among individual responses and led to issues associated with the rigour and integrity of the data (Boritz, 2005). However, training the research team, both theoretically and practically with a piloting phase, aimed to ensure appropriate tool development and data collection (Gilson *et al.*, 2011), and ultimately to improve the faithfulness of information we collected. In addition, ‘internal reliability’ (Bryman, 2008) was enhanced by interviewers working together to ensure rigour and consistency in data analysis and agreement about the presentation of research findings.

Finally, the reliability of coding is a further potential area of concern in our research. Coding must be done in a consistent manner between coders (*inter-coder reliability*), and each coder must be consistent over time (*intra-coder reliability*; Bryman, 2008). In our study, after each step of coding, the research team discussed to reach to an agreement on the coding and categorization.

## Conclusion

In contrast with the IE that found no evidence of an impact of PBF on improving the availability of EMs except for family planning, our findings suggest that the PBF intervention improved the perceived availability of EMs in three regions in Cameroon. By cautiously examining these potential effects, we have identified several underlying pathways that include more autonomy of HFs, the enforced regulation from the DMT, the greater accountability of the pharmacy attendant and supply system liberalization. However, our results suggest that there is no linear trail as many potential mediating factors are central to comprehensively understand the dynamic of the implementation processes around PBF. Contextual, organizational and leadership factors might explain heterogeneity in performance between HFs, suggesting that facilities with good geographical accessibility, that are well equipped, and where managers play active leadership, may have succeeded under the programme to take advantages of autonomization and liberalization processes.

Drawing from our findings, the present study has some policy implications that include the following recommendations:

- Communicating and formalizing the content of ‘autonomy’ and ‘liberalization’ in order to engage front-line health workers commitment in their implementation;
- Strengthening the role of facility managers to translate the innovative policy into desired interventions;
- Putting in place mechanisms to promote equity in order to avoid worsening pre-existing inequalities with the pharmacy autonomization;
- Paying attention to reinforcing regulation and quality assurance at the regional level, as opening the supply system to private wholesalers generates some concerns about the quality of drugs.

*Ethics approval:* The study protocol was reviewed and approved by the Cameroon National Ethics Committee for Human Health Research (N0 2015/02/549/CE/CNERSH/SP) and the WHO Research Ethics Review Committee (RBF2014–395). Administrative authorization was granted by the Cameroonian Ministry of Health (D30-298 L/MINSANTE/SG/DROS/CRSPE/CEA2) prior to data collection. All respondents provided verbal or written informed consent before interviews. To ensure the confidentiality and anonymity of the data, we replaced participants’ names with codes.

## Supplementary Material

czz084_Supplementary_Appendix
