# Supplementary material for: How does performance-based financing affect the availability of essential medicines in Cameroon? A qualitative study
Source: Health Policy Plan. 2019 Dec 9;34(Suppl 3):iii4–iii19. doi: 10.1093/heapol/czz084 (PMC6901074; doi:10.1093/heapol/czz084)
Supplement: czz084_Supplementary_Appendix [file czz084_supplementary_appendix.zip › czz084-suppl_data/Supplementary Appendix S2.docx]

**Appendix2: Terms of reference for the district health service in performance based financing**

- Create the enabling environment to integrate the Performance Based Financing approach in to the management of health District activities.
- Sensitize the health staff of the entire District as well as the members of the District Health and the respective communities served by the District Health service on the importance of PBF and their effective implication in its implementation.
- Support the autonomization process of health facilities
- In collaboration with the Fund Holder Agency, develop the three-monthly action plans for the service to be carried out within the context of the implementation of PBF and based on the health development plan of the District;
- Submit the action plan to AEDES/IRESCO for approval before implementation
- Submit a quarterly activity report (with supporting documents) to AEDES/IRESCO
- In collaboration with the RDPH and AEDES/IRESCO staff, carry out quarterly quality assessment of the Health facilities (health centers and hospitals) of the Health District
- Contribute in the training of frontline health facility staff (IHC and Hospitals) and community members on PBF related modules.

| **Activities** | **Month1** | | | | **Month2** | | | | **Month3** | | | | **Comments** | | | | **Responsible** |
| --- | --- | --- | --- | --- | --- | --- | --- | --- | --- | --- | --- | --- | --- | --- | --- | --- | --- |
| **Objective 1** Create the enabling environment to integrate the Performance Based Financing approach in to the management of health District activities | | | | | | | | | | | | | | | | | |
| 1. welcome and introduce PBF verificators to other staff of the District Health Service | X |  |  |  |  |  |  |  |  |  |  |  |  |  |  |  | DHT |
| 2.Allocate working space for the PBF verificators at the District Health service | X |  |  |  |  |  |  |  |  |  |  |  |  |  |  |  | DHT |
| 3 Assist the PBF verificators to settle in Health District | X |  |  |  |  |  |  |  |  |  |  |  |  |  |  |  | DHT |
| **Objective 2** Sensitize the health staff of the entire District as well as the members of the District Health for Health and the respective communities served by the District Health service on the importance of PBF and their effective implication in its implementation | | | | | | | | | | | | | | | | | |
| 1.Carry out advocacy with the SDO on PBF | X |  |  |  |  |  |  |  |  |  |  |  |  |  |  |  | DMO |
| 2.Continue to keep inform other administrative authorities and updated on all the activities of PBF in the District. | X | X | X | X | X | X | X | X | X | X | X | X | X | X | X | X | DHT |
| 3.Discuss about PBF during monthly District coordination meetings. |  |  |  | X |  |  |  | X |  |  |  | X |  |  |  | X | DHT |
| 4 Hold staff meetings during quality verifications to continue sensitize and give them feed back |  |  | X |  |  |  | X |  |  |  |  | X |  |  | X |  | DHT |
| 5 Organize District General Assembly meeting to sensitize them on PBF |  |  |  |  |  |  |  |  |  |  |  | X |  |  |  |  | DHT |
| 6. Organize in collaboration with the Regional Delegation a launching ceremony to explain and sensitize the population and other related sectors on the principles of PBF. |  |  |  |  | X |  |  |  |  |  |  |  |  |  |  |  | DHT |
| 7. Organize monthly radio talks radio talks on Boyo Community Radio on PBF and other health related issues. | X |  |  |  | X |  |  |  |  | X |  |  |  | X |  |  | DHT |
| **Objective 3** Support the autonomization process of health facilities | | | | | | | | | | | | | | | | | |
| 1. Support health facilities to open their bank account | X | X | X |  |  |  |  |  |  |  |  |  |  |  |  |  |  |
| 2. Train the managers of health facilities on resource management | X | X | X |  |  |  |  | X | X | X |  |  |  |  |  |  |  |
| 3. Train the managers of health facilities and the pharmacy attendants on pharmacy management | X | X | X |  |  |  |  | X | X | X |  |  |  |  |  |  |  |
| 4. Provide health facilities with the list of accredited drug wholesalers in the region | X | X | X |  |  |  |  |  | X | X | X |  |  |  |  |  |  |
| **Objective 4** In collaboration with the Fund Holder Agency, develop the three monthly action plans for the service to be carried out within the context of the implementation of PBF and based on the health development plan of the District | | | | | | | | | | | | | | | | | |
| 1.Drawing of first action plan |  |  |  | X |  |  |  |  |  |  |  |  |  |  |  |  | DHT |
| **Objective 5** Submit the action plan to AEDES/IRESCO for approval before implementation | | | | | | | | | | | | | | | | | |
| 1.Action plan submitted before the signing of the contract |  |  |  | X |  |  |  |  |  |  |  |  |  |  |  |  | DHT |
| **Objective 6** Submit a quarterly activity report (with supporting documents) to AEDES/IRESCO | | | | | | | | | | | | | | | | | |
| 1. Summit report on training of chief of health units and chairmen of health committees | X |  |  |  |  |  |  |  |  |  |  |  |  |  |  |  | DHT |
| 2. Summit report on the first verification and establishment of business plans |  |  |  | X |  |  |  |  |  |  |  |  |  |  |  |  | DHT |
| 3. Carry out monthly supervision of all health centers |  |  |  | X |  |  |  | X |  |  |  | X |  |  |  | X | DHT |
| 4. Carryout monthly evaluation of the business plans of all health units |  |  |  | X |  |  |  | X |  |  |  | X |  |  |  | X | DHT |
| 5. Summit monthly reports of supervision and evaluation of health units |  |  |  | X |  |  |  | X |  |  |  | X |  |  |  | X | DHT |
| 6. Give oral and written feedback to all health units as concerns their performances |  |  |  | X |  |  |  | X |  |  |  | X |  |  |  | X | DHT |
| **Objective 7**: In collaboration with the RDPH and AEDES/IRESCO staff, carry out quarterly quality assessment of the Health facilities (health centers and hospitals) of the Health District | | | | | | | | | | | | | | | | | |
| 1.Carry out Quality assessment of all health units in T and C1 |  |  | X |  |  |  |  |  |  |  |  |  |  |  |  |  | DHT |
| 2. Assist Health units in establishing their Business plans |  |  | X |  |  |  |  |  |  |  |  |  |  |  |  |  | DHT |
| 3. Organise a preparatory meeting for the signing of the various business plans of the health units |  |  | X |  |  |  |  |  |  |  |  |  |  |  |  |  | DHT |
| 4. Organise in collaboration with the RDPH and the FHA the signing of contracts by the various health units |  |  | X |  |  |  |  |  |  |  |  |  |  |  |  |  | DHT |
| **Objective 8:** Contribute in the training of frontline health facility staff (IHC and Hospitals) and community members on PBF related modules | | | | | | | | | | | | | | | | | |
| 1. Training of managers of Health units and chairpersons of management committees | X |  |  |  |  |  |  |  |  |  |  |  |  |  |  |  | DHT |
| 2. Organize on the spot trainings during monthly supervision of Health units as the need arises |  |  |  | X |  |  |  | X |  |  |  | X |  |  |  | X | DHT |
