# Supplementary material for: How does performance-based financing affect the availability of essential medicines in Cameroon? A qualitative study
Source: Health Policy Plan. 2019 Dec 9;34(Suppl 3):iii4–iii19. doi: 10.1093/heapol/czz084 (PMC6901074; doi:10.1093/heapol/czz084)
Supplement: czz084_Supplementary_Appendix [file czz084_supplementary_appendix.zip › czz084-suppl_data/Supplementary Appendix S1.docx]

**Appendix1: Indicators of the health centers’ Quality Assessment Checklist related to Essential Drugs**

| 1. **FAMILY PLANNING** | **Protocol respected** | **Protocol**  **not respected** |
| --- | --- | --- |
| 1. Security Stock for oral and injectable contraceptives available   *10 000 inhabitants = 147 doses DEPO and 36 doses pills* | 3 | 0 |

| 1. **DRUGS AND SUPPLIES MANAGEMENT** | **Protocol respected** | **Protocol**  **not respected** |
| --- | --- | --- |
| 1. The staff indicate the security stock on the stock cards as = *the average monthly consumption rate /2*   *Stock in the cards corresponds to the physical stock (real stock)*   - *The evaluator takes a sample of three drugs* | 5 | 0 |
| 1. The health staff (health facility) has access to the accredited distribution (sales) centers for drugs, equipment and supplies known to the health district | 1 | 0 |
| 1. The drugs are well arranged and well kept.   *Tidy room and well aerated with cupboards and shelves and drugs arrange according to class and alphabetical order* | 2 | 0 |
| 1. The main pharmacy store (warehouse) supplies the facility sales point on a daily basis as per requested needs   *- The evaluator verifies if the quantities requested by the sales agent (clerk) correspond to the quantities supplied (check the request slip co-signed by the two parties).* | 2 | 0 |
| 1. Absence of expired drugs or drugs with falsified tickets   *- The evaluator draws 3 drugs and 2 supplies by chance :*  *- Expired drugs well separated from the rest of the drug*  *- A monthly inventory of expired drugs done and sent to the District Health Service (reception note available) or destroyed according to norms with a report written* | 2 | 0 |
| 1. Drugs sales point in the facility (pharmacy):    - *Drugs dispensed in sachets or small bags.*    - *Tablets manipulated (dispensed using a spoon*    - *Availability of portable water for taking first dose of drugs in the health* | 3 | 0 |

| 1. **TRACER DRUGS** ***Security Stock =*** ***Monthly average consumption (MAC)/ 2*** | **Available Yes**  **> MAC / 2** | **Available NO**  **< MAC / 2** |
| --- | --- | --- |
| 1. Amoxicillin caps /tabs 500 mg | 1 | 0 |
| 1. Amoxicillin syrup 250 mg/ 5ml | 1 | 0 |
| 1. Artesunate tabs 50 mg – amodiaquine 200 mg | 1 | 0 |
| 1. Cotrimoxazol tabs 480 mg | 1 | 0 |
| 1. Diazepam 10 mg / 2ml – injectable | 1 | 0 |
| 1. Iron – folic acid 200 mg + 25 mg | 1 | 0 |
| 1. Mebendazol tabs 100 mg | 1 | 0 |
| 1. Methergine/syntocinone amp 10 Units | 1 | 0 |
| 1. Metronidazol tabs 250 mg | 1 | 0 |
| 1. Paracetamol tabs 500 mg | 1 | 0 |
| 1. Quinine tabs 300 mg and quinine injectable | 1 | 0 |
| 1. ORS / oral sachet | 1 | 0 |
| 1. Sterile gloves | 1 | 0 |
| 1. Sterile gauze | 1 | 0 |
| 1. glucose Solution 5% | 1 | 0 |

| 1. **VACCINATION** | **Protocol respected** | **Protocol**  **not respected** |
| --- | --- | --- |
| 1. **Vaccines not out of stock (**DPT+HepB+Hip, BCG, Measles + Yellow fever, OPV, TT)   *- Stock cards available and up-to-date*  *- The evaluator verifies the physical stock in the fridge which has to be correspond to the theoretical stock* | 1 | 0 |
| 1. **Syringes available**   *- auto disable – at least 30*  *- Solvent – at least 3* | 1 | 0 |

| **Total** | **35** |  |
| --- | --- | --- |
