# Supplementary material for: How does performance-based financing affect the availability of essential medicines in Cameroon? A qualitative study
Source: Health Policy Plan. 2019 Dec 9;34(Suppl 3):iii4–iii19. doi: 10.1093/heapol/czz084 (PMC6901074; doi:10.1093/heapol/czz084)
Supplement: czz084_Supplementary_Appendix [file czz084_supplementary_appendix.zip › czz084-suppl_data/Supplementary Appendix S3.docx]

**Appendix3: Terms of reference for the regional delegation of health in performance based financing**

- In collaboration with the Fund Holder Agency, develop the three-monthly action plans for the Regional Delegation to be carried out within the context of the implementation of PBF and based on the health development plan of the region;
- Organize and oversee the quarterly quality assessments of the District and assimilated Hospitals implementing PBF at the end of the quarter
- Support the autonomization process of health facilities
- Create the Regional PBF Task Force and organize the steering meeting
- Submit a quarterly activity report to AEDES/IRESCO

| **Activities** | **Month1** | | | | **Month2** | | | | **Month3** | | | | **Comments** | | | | **Responsible** |
| --- | --- | --- | --- | --- | --- | --- | --- | --- | --- | --- | --- | --- | --- | --- | --- | --- | --- |
| **Objective 1** In collaboration with the Fund Holder Agency, develop the three-monthly action plans for the Regional Delegation to be carried out within the context of the implementation of PBF and based on the health development plan of the region | | | | | | | | | | | | | | | | | |
| 1.Drawing of first action plan | X |  |  |  |  |  |  |  |  |  |  |  |  |  |  |  | RD |
| **Objective 2** Organize and oversee the quarterly quality assessments of the District and assimilated Hospitals implementing PBF at the end of the quarter | | | | | | | | | | | | | | | | | |
| 1.Carry out Quality assessment of all hospitals |  |  |  |  |  |  |  |  |  |  |  | X |  |  |  |  | RD |
| **Objective 3** Support the liberalization of EMs supply | | | | | | | | | | | | | | | | | |
| 1. Conduct quality-assurance of EMs providers in the region | X | X | X |  |  |  |  |  |  |  |  |  |  |  |  |  | RD |
| 2. Draw-up the list of the accredited wholesalers in the region | X | X | X |  |  |  |  |  |  |  |  |  |  |  |  |  | RD |
| 4. Provide district health services with the list of accredited drug wholesalers in the region | X | X | X |  |  |  |  |  | X | X | X |  |  |  |  |  | RD |
| **Objective 4** Create the Regional PBF Task Force and organize the steering meeting | | | | | | | | | | | | | | | | | |
| 1. Create the Regional PBF Task Force |  |  |  | X |  |  |  |  |  |  |  |  |  |  |  |  | RD |
| 2. Organize the regional PBF steering committee meeting |  |  |  |  |  |  |  |  |  |  |  |  |  |  |  |  | RD |
| **Objective 5** Submit a quarterly activity report (with supporting documents) to AEDES/IRESCO | | | | | | | | | | | | | | | | | |
| 3. Carry out monthly supervision of all district hospitals |  |  |  | X |  |  |  | X |  |  |  | X |  |  |  | X | RD |
| 4. Carryout monthly evaluation of the business plans of all district hospitals |  |  |  | X |  |  |  | X |  |  |  | X |  |  |  | X | RD |
| 5. Summit monthly reports of supervision and evaluation of district hospitals |  |  |  | X |  |  |  | X |  |  |  | X |  |  |  | X | RD |
| 6. Give oral and written feedback to all district hospital as concerns their performances |  |  |  | X |  |  |  | X |  |  |  | X |  |  |  | X | RD |
